# Is conformation a fundamental descriptor in QSAR? A case for halogenated anesthetics

**DOI:** 10.3762/bjoc.12.76

**Published:** 2016-04-21

**Authors:** Maria C Guimarães, Mariene H Duarte, Josué M Silla, Matheus P Freitas

**Affiliations:** 1 Department of Chemistry, Federal University of Lavras, P. O. Box 3037, 37200-000, Lavras, MG, Brazil

**Keywords:** conformational analysis, isoflurane, QSAR, theoretical calculations, volatile anesthetics

## Abstract

An intriguing question in 3D-QSAR lies on which conformation(s) to use when generating molecular descriptors (MD) for correlation with bioactivity values. This is not a simple task because the bioactive conformation in molecule data sets is usually unknown and, therefore, optimized structures in a receptor-free environment are often used to generate the MD´s. In this case, a wrong conformational choice can cause misinterpretation of the QSAR model. The present computational work reports the conformational analysis of the volatile anesthetic isoflurane (2-chloro-2-(difluoromethoxy)-1,1,1-trifluoroethane) in the gas phase and also in polar and nonpolar implicit and explicit solvents to show that stable minima (ruled by intramolecular interactions) do not necessarily coincide with the bioconformation (ruled by enzyme induced fit). Consequently, a QSAR model based on two-dimensional chemical structures was built and exhibited satisfactory modeling/prediction capability and interpretability, then suggesting that these 2D MD´s can be advantageous over some three-dimensional descriptors.

## Introduction

Quantitative structure–activity relationship (QSAR) studies try to find a correlation between chemical structures and the corresponding bioactivities by means of molecular descriptors (MD´s). In this way, molecular architecture and substitution patterns in a series of congeneric molecules are described by calculable or empirical data having some relationship with biological activity, becoming the technique useful for understanding the action mechanism of related drugs, as well as to drive the synthesis of new drug like compounds.

Since the milestone work by Hansch and Fujita [1], a variety of MD´s have been developed to improve the correlation of chemical structures with bioactivity, ranging from hydrophobicity to three-dimensional descriptors [2]. Indeed, the most popular QSAR methods are based on molecular descriptors generated from 3D molecular structures, such as the widely used techniques based on molecular field analysis [3–4]. The problem with most 3D-QSAR methods is that the bioactive conformation of the compounds in a data set is usually unknown and, therefore, geometry optimization is carried out in a receptor-free environment to generate the molecular structure and, subsequently, the 3D MD´s. While molecular conformation in vacuum is governed by intramolecular interactions, the bioconformation is ruled by enzyme induced-fit; consequently, optimized and bioactive geometries are probably different to each other and, to obtain insight on the action mechanism of a drug and substituent effects, MD´s should not be generated over geometries optimized in a receptor-free environment. Efforts have been made to attenuate the drawback of using a conformation that is possibly wrong, e.g., by using average conformations, ensemble and multidimensional methods [5–8], but the risk of chemical–biological misinterpretation remains. Receptor-dependent QSAR methods have also been developed [9], but these are mostly complementary and are aimed at corroborating and/or rationalizing the results provided by the regression models, since the docking methodology itself provides intermolecular energies and docking scores that correlate with bioactivity. On the other hand, despite not encoding conformational information, 2D-QSAR can incorporate other stereochemical properties and also account for group sizes, substituent type and 2D shape of molecules. These have shown to be sufficient parameters to obtain satisfactory correlation with bioactivity data and valuable understanding on structural requirements for drug development.

Multivariate Image Analysis applied to QSAR (MIA-QSAR) is a genuine 2D method, since descriptors are pixels of molecular projections drawn as black and white wireframes [10] and, more recently, as colored 2D-images (chemical structures) with spheres representing atoms with sizes proportional to the respective van der Waals radii [11]. The variance in the atom color and coordinate of pixels in the images (the structural variance) explains the changes in the bioactivities block. Thus, the MIA-QSAR method is an appropriate approach to probe the validity of 2D-QSAR methods when the molecules in a data set undergo rotational isomerization.

In order to test our hypothesis, the biological activities of a set of volatile halogenated anesthetics were modelled using the recent version of the MIA-QSAR method. In addition, the suitability of applying 3D information obtained from high level calculations (in a receptor-free environment) in QSAR modelling was evaluated using a comparative study of the optimized and bioactive conformations of the fluorinated anesthetic isoflurane, which binds to a 4-helix bundle protein (apoferritin) [12] and to the integrin LFA1 enzyme [13].

## Results and Discussion

### Conformational analysis

**Figure 1:**
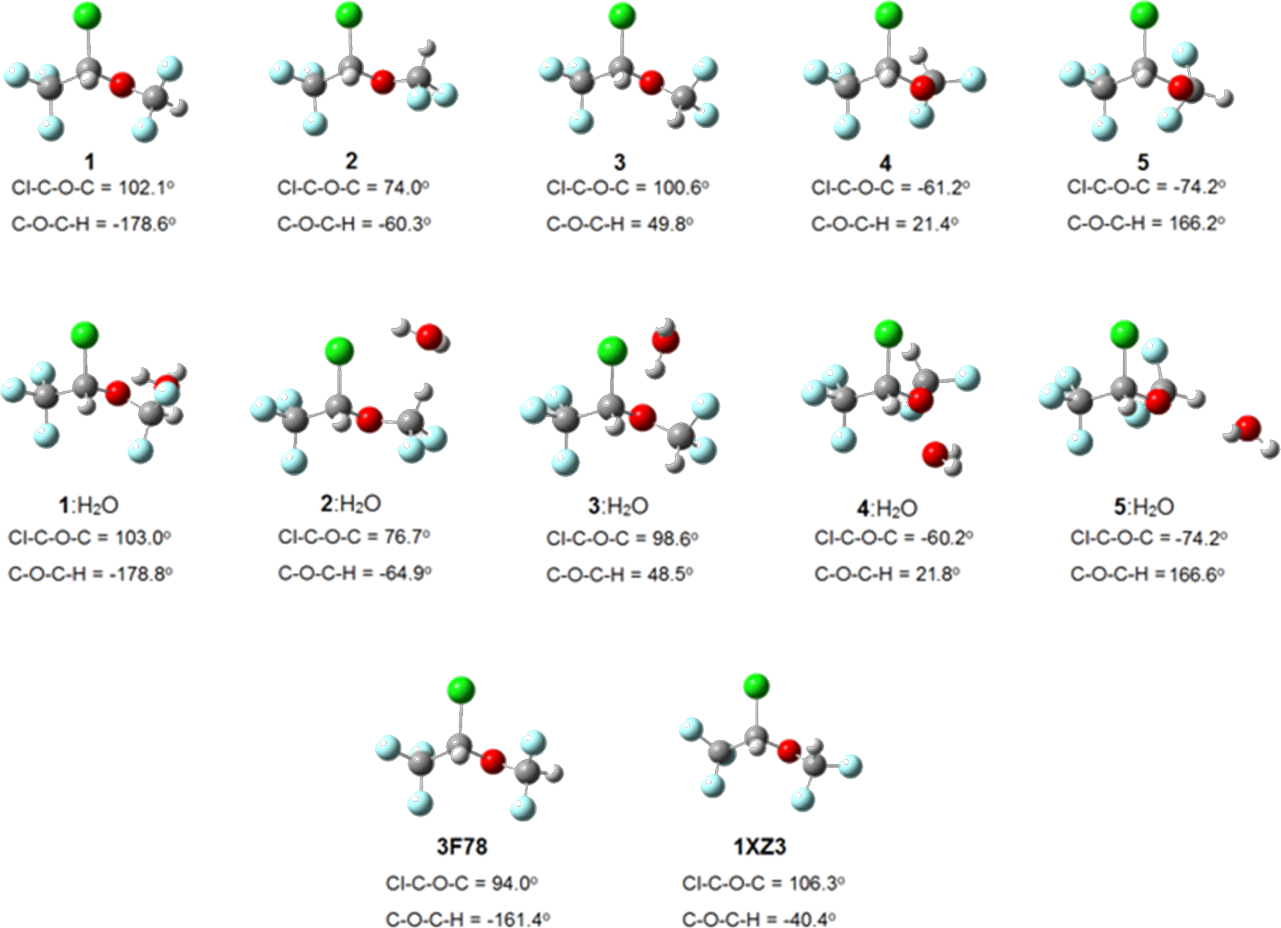
Optimized structures of isoflurane at the ωB97X-D/6-311++g(d,p) level (gas phase and explicit water) and the experimental bioconformations 3F78 and 1XZ3.

2-Chloro-2-(difluoromethoxy)-1,1,1-trifluoroethane (isoflurane) undergoes rotational isomerization around two dihedral angles (Cl–C–O–C and C–O–C–H) and, considering three limit orientations for each of them (*gauche*, *gauche'* and *anti*), nine conformations are possible for isoflurane. However, geometry optimization at the MP2/6-311++g(d,p) and ωB97X-D/6-311++g(d,p) (a DTF method which includes dispersion effects) levels converged to five energy minima for the gas phase, implicit solvents (cyclohexane, DMSO and water, using the polarizable continuum model), and using one explicit water molecule as solvent to mimic a physiological medium (Figure 1).

The results for the gas phase are in agreement with previous calculations [14] and microwave experiments [15], where five conformations were found, but only three could be experimentally detected, due to their lower energies. These correspond to conformers **1**, **2** and **3** of Table 1, the most stable ones in the gas phase and implicit solution. It is worth mentioning that, according to our calculations, the solvent has a little effect on the conformer populations, such as in enflurane [16], suggesting that intramolecular interactions govern the conformational equilibrium in a biological-free environment. Indeed, Lesarri et al. [15] pointed out that anomeric effects owing to donor–acceptor (LP → σ*) interactions are responsible for the conformational preference of isoflurane. Despite the contribution from specific hyperconjugative interactions representing the anomeric effects (mainly LP_O_ → σ*_CF_, Table 2), our natural bond orbital (NBO) analysis indicates that the main conformers **1** and **2** are less favored by electronic delocalization than **3**, **4** and **5**. Since the overall energy of a system is a composition of repulsive Lewis (steric and electrostatic) and attractive non-Lewis-type (hyperconjugation) contributions, the main factor governing the conformational stabilization of isoflurane comes from the more classical steric and dipolar interactions, likewise sevoflurane reported earlier [17].

**Table 1:** Relative electronic/Gibbs free energies (kcal mol^−1^, % Gibbs population in parenthesis) for the conformers of isoflurane.^a^

Medium	**1**	**2**	**3**	**4**	**5**

DFT					

Gas	0.0/0.0 (64)	0.7/0.4 (32)	2.5/1.9 (3)	2.3/2.4 (1)	5.3/6.2 (0)
C_6_H_12_	0.0/0.0 (59)	0.7/0.3 (36)	2.0/1.6 (4)	2.3/2.6 (1)	5.3/6.2 (0)
DMSO	0.0/0.0 (37)	0.4/0.2 (31)	0.8/0.1 (31)	2.3/2.6 (1)	5.1/6.0 (0)
H_2_O	0.0/0.0 (37)	0.4/0.2 (26)	0.7/0.0 (37)	2.3/2.5 (0)	5.1/6.0 (0)
H_2_O_explicit_	0.0/0.0 (85)	1.0/1.1 (14)	4.6/3.8 (0)	1.7/2.5 (1)	6.3/6.8 (0)

MP2					

Gas	0.0/0.0 (71)	0.8/0.6 (26)	2.5/1.9 (3)	2.8/3.0 (0)	5.5/6.4 (0)
C_6_H_12_	0.0/0.0 (68)	0.8/0.6 (25)	2.1/1.3 (8)	2.9/3.2 (0)	5.4/6.1 (0)
DMSO	0.0/0.0 (51)	0.4/0.3 (31)	0.9/0.6 (18)	2.8/3.1 (0)	5.3/6.0 (0)
H_2_O	0.0/0.0 (54)	0.8/0.5 (23)	0.8/0.5 (23)	2.8/3.1 (0)	5.3/6.0 (0)
H_2_O_explicit_	0.0/0.0 (88)	1.2/1.2 (12)	4.6/3.8 (0)	2.2/3.0 (0)	6.5/6.9 (0)

^a^The converged geometries did not exhibit imaginary frequency and the standard Gibbs free energies were theoretically calculated under the conditions 1.00 atm and 298.15 K, including electronic and thermal corrections.

**Table 2:** Anomeric interactions and relative Lewis and non-Lewis contributions (kcal mol^−1^) for the conformers of isoflurane, in the gas phase at the ωB97X-D/6-311++g(d,p) level.

Conformer	n_O_ → σ^*^_CF_	n_O_ → σ^*^_CCl_	n_O_ → σ^*^_CCF3_	n_O_ → σ^*^_C2H_	n_O_ → σ^*^_C1H_	*E*_Lewis_	*E*_non-Lewis_

**1**	15.5 + 2.4 + 15.9 + 2.6	17.6	4.2	3.9 + 1.1	3.7	0.0	0.3
**2**	15.4 + 2.4 + 7.3	1.0 + 21.3	3.4 + 0.8	5.4 + 2.7	0.6 + 7.7	0.4	0.0
**3**	7.0 + 0.8 + 20.1	19.2	1.8 + 4.9	4.4 + 1.0	2.3 + 5.1	10.9	8.7
**4**	6.1 + 5.3 + 18.6	3.1 + 18.1	2.1 + 9.4	4.6	3.3 + 1.7	6.9	4.9
**5**	2.0 + 17.4 + 3.6 + 11.6	1.1 + 19.1	3.8 + 6.4	4.4 + 0.6	4.0	18.1	13.1

It has been proposed that intramolecular CH∙∙∙FC hydrogen bond drives the conformational preference of enflurane [18]. In order to check if such an interaction operates in isoflurane, QTAIM (Quantum Theory of Atoms in Molecules) calculations were performed for **1–5**. The molecular graphs of Table 3 do not exhibit F∙∙∙H or Cl∙∙∙H bond paths (only weak nonbonding interactions are observed for the high energy conformers **4** and **5**). According to Koch and Popelier [19], the following QTAIM parameters, obtained by integration over the atomic basins of the hydrogen atoms participating in hydrogen bonds, should be observed to characterize hydrogen bonds along with a bond path: loss of atomic charge (*q*), decreased first dipole moment (*M*_1_), decreased atomic volume (*V*) and increased atomic energy (*E*). Since these criteria do not vary significantly among the conformers (either when H approaches to Cl and F or not), we can confirm that hydrogen bonding is not a decisive interaction for the conformational equilibrium of isoflurane.

**Table 3:** Atomic properties (a.u.) obtained by QTAIM to characterize hydrogen bonds in isoflurane.

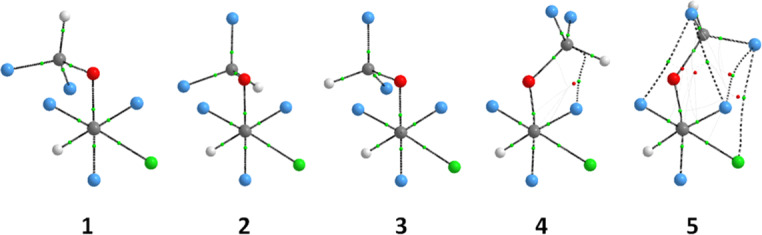				

Conformer	*q* (H)	*M*_1_ (H)	*V* (H)	*E* (H)

**1**_H(CCl)/H(CF2)_	+0.126/+0.126	+0.125/+0.127	+39.37/+41.03	−0.583/−0.589
**2**_H(CCl)/H(CF2)_	+0.122/+0.112	+0.127/+0.133	+40.11/+41.94	−0.584/−0.591
**3**_H(CCl)/H(CF2)_	+0.097/+0.093	+0.135/+0.136	+42.15/+42.84	−0.592/−0.599
**4**_H(CCl)/H(CF2)_	+0.128/+0.119	+0.127/+0.127	+40.80/+38.93	−0.584/−0.591
**5**_H(CCl)/H(CF2)_	+0.123/+0.118	+0.128/+0.129	+40.66/+41.30	−0.588/−0.593

Using a solvation model with explicit water, where specific solute–solvent interactions take place, conformer **1** is again the most stable form, suggesting that implicit solvation describes satisfactorily the actual conformational isomerism. However, is there an environment capable of overcoming intramolecular interactions and then changing the conformational preference of isoflurane? While comparison of **1** with the bioconformation of isoflurane bound to the integrin LFA1 enzyme (PDB code: 3F78 [13]) reveals small differences in the Cl–C–O–C and C–O–C–H dihedral angles (±8° and 17°, respectively), the isoflurane structure bound to apoferritin (PDB code: 1XZ3 [9]) does not match any optimized conformer (Figure 1). So, does it make sense to use enzyme-free optimized conformations to obtain biochemical insights from 3D-QSAR? The following MIA-QSAR modeling can help to elucidate this question and to support the use of 2D approaches with the aim for obtaining biochemical information.

### MIA-QSAR of anesthetic haloethers

**Figure 2:**
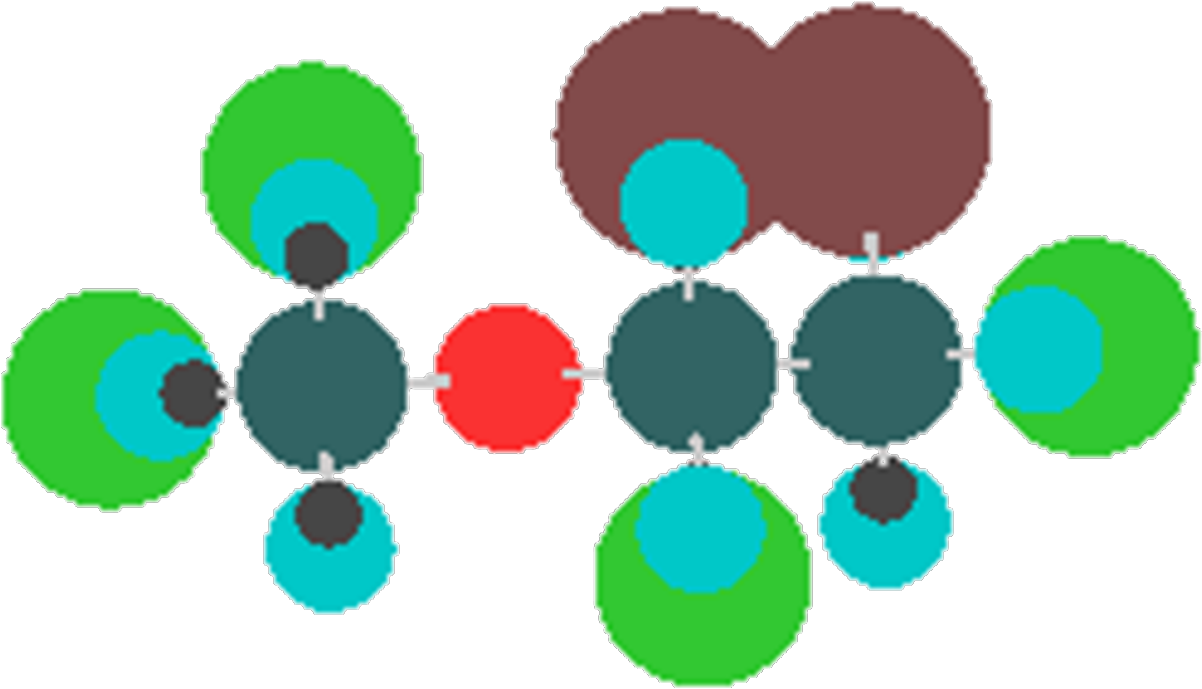
Superimposed chemical structures used to generate the MIA descriptors with atoms colored according to the corresponding Pauling’s electronegativity.

MIA-QSAR is a genuine 2D technique in the sense that it takes into account 2D projections of chemical structure images to generate MD's. Each compound of a congeneric series is aligned to one another by a congruent motif and, therefore, the variable substructure along the series explains the variance in the bioactivities block. The variance in the chemical structures is captured by the different coordinates of the pixels composing the images (Figure 2 shows the superimposed images used in this work to illustrate the structural variance). Pixels also vary in color, depending on the atom to which they refer. Since pixel values are a summation of RGB (red-green-blue) components and each channel is numerically equivalent to 255, the whole spectrum of colors can vary from 0 (black) to 765 (white). The pixel value (atom color) can be managed according to atomic properties and, therefore, each different atom in the series of 25 haloethers of Table 4 [20–21] was colored according to the respective Pauling's electronegativity (ε) scale, since polar properties should help to modulate their mode of interaction with an enzyme (see Table 5 for correspondence of ε with pixel values and approximate colors). Another useful parameter ruling bioactivity is the steric effect, which was accounted for by representing atoms in the molecules as colored spheres with sizes proportional to the corresponding van der Waals radii. The molecules were constructed using the GaussView program [22] and each 2D projection (each image, molecule) with 342 × 300 pixels dimension was unfolded to a row vector. Combination of the 25 images yielded a data matrix, which was regressed against the bioactivities column vector (pMAC, the negative logarithm of the partial pressure capable of suppressing the movement in response to noxious stimuli in 50% of rats) using partial least squares (PLS) regression. The MIA-QSAR model was constructed over 19 training set compounds and externally validated using the remaining 6 compounds (**7**, **8**, **9**, **15**, **17** and **19**), which were chosen using Kennard–Stone sampling.

**Table 4:** Data set of anesthetic haloethers and the corresponding experimental pMAC values.

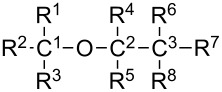			

Compound	Chemical formula	Structural formula	pMAC

**1**	C_3_Cl_3_F_5_O	CClF_2_OCCl_2_F	0.88
**2**	C_3_Cl_3_F_5_O	CClF_2_OCF_2_CCl_2_F	0.73
**3**	C_3_Cl_3_F_5_O	CCl_2_FOCF_2_CClF_2_	0.74
**4**	C_3_ClF_6_HO	CClF_2_OCFHCF_3_	0.54
**5**	C_3_ClF_6_HO	CF_2_HOCClFCF_3_	0.49
**6**	C_3_ClF_6_HO	CF_2_HOCF_2_CClF_2_	0.22
**7**	C_3_ClF_5_H_2_O	CClF_2_OCH_2_CF_3_	0.54
**8**	C_3_ClF_5_H_2_O	CF_2_HOCClHCF_3_	1.84
**9**	C_3_ClF_5_H_2_O	CF_2_HOCF_2_CClFH	1.66
**10**	C_3_Cl_2_F_5_HO	CClF_2_OCClHCF_3_	1.31
**11**	C_3_Cl_2_F_5_HO	CF_2_HOCCl_2_CF_3_	1.01
**12**	C_3_Cl_2_F_5_HO	CClF_2_OCF_2_CClFH	1.52
**13**	C_3_Cl_2_F_5_HO	CF_2_HOCF_2_CFCl_2_	1.04
**14**	C_3_Cl_2_F_2_H_4_O	CH_3_OCF_2_CHCl_2_	2.57
**15**	C_3_F_7_HO	CF_2_HOCF_2_CF_3_	−0.75
**16**	C_3_F_7_HO	CF_3_OCFHCF_3_	−0.29
**17**	C_3_F_6_H_2_O	CF_2_HOCFHCF_3_	1.11
**18**	C_3_F_5_H_3_O	CF_2_HOCH_2_CF_3_	0.96
**19**	C_3_F_5_H_3_O	CFH_2_OCFHCF_3_	1.33
**20**	C_3_F_5_H_3_O	CFH_2_OCF_2_CF_2_H	1.37
**21**	C_3_ClF_3_H_4_O	CH_3_OCF_2_CClFH	1.80
**22**	C_3_BrClF_5_HO	CF_2_HOCBrClCF_3_	1.82
**23**	C_3_BrClF_5_HO	CF_2_HOCF_2_CBrClF	1.82
**24**	C_3_BrF_5_H_2_O	CF_2_HOCBrHCF_3_	2.28
**25**	C_3_BrF_3_H_4_O	CH_3_OCF_2_CBrFH	2.16

**Table 5:** Correspondence of Pauling's electronegativity with atomic colors and respective pixel values (as a combination of RGB components) used in the MIA-QSAR model.

Atom	ε	Color	Pixel value

H	2.1	charcoal	210
C	2.5	teal	250
O	3.5	red	350
F	4.0	turquoise	400
Cl	3.0	green	300
Br	2.8	maroon	280
chemical bond	–	grey	612
blank space	–	white	765

**Figure 3:**
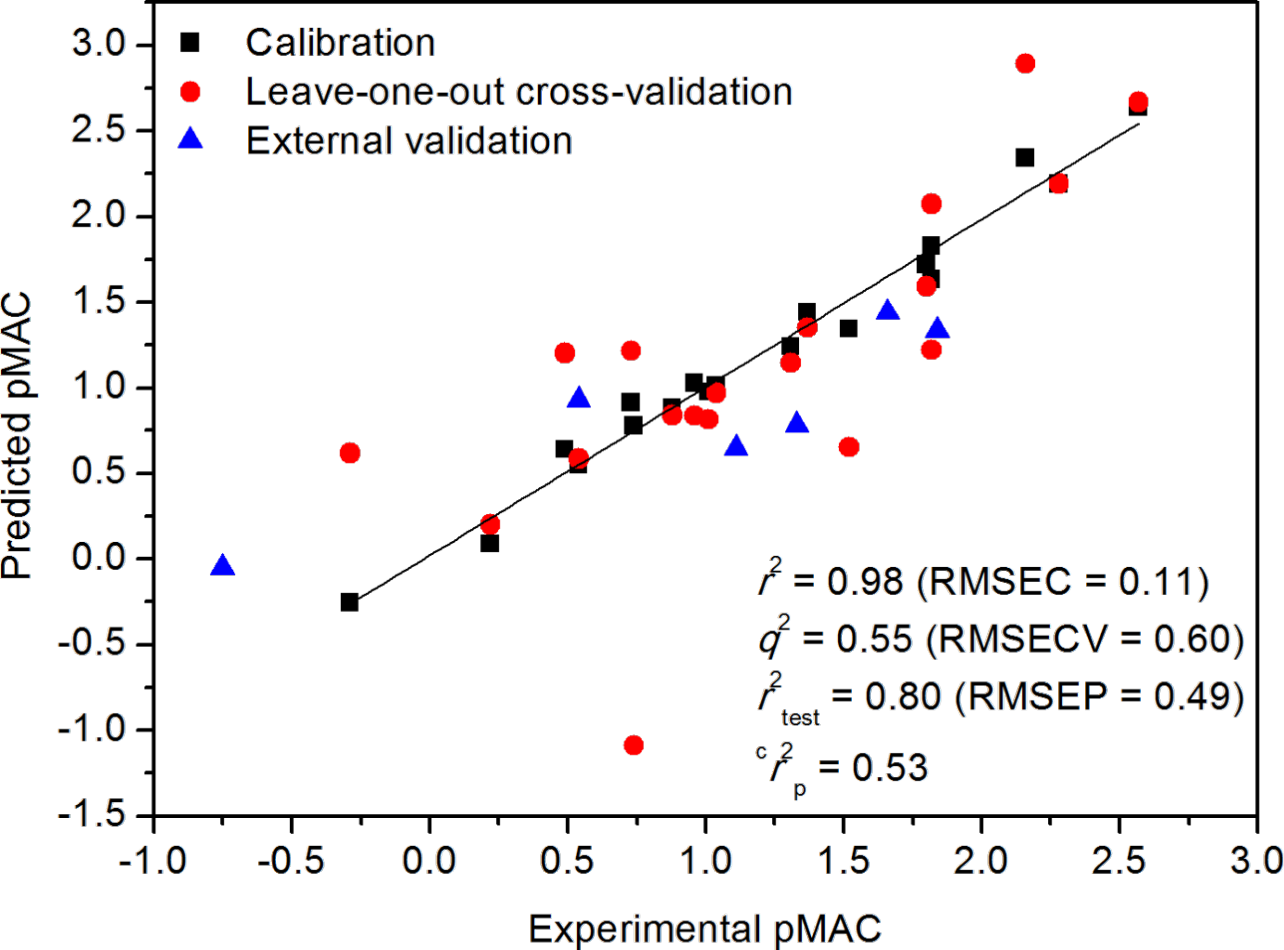
Plot of actual vs predicted pMAC obtained from the MIA-QSAR model.

A good correlation between MIA descriptors and the pMAC values for the series of anesthetic haloethers indicates that 2D chemical structures encode biological activities. A previous study using log *P* (the octanol/water partition coefficient) as descriptor showed a good correlation with pMAC [21], but the resulting model was not validated. Thus, the present study provided an internal (through leave-one-out cross-validation) and external validation to attest the reliability of the MIA-QSAR model. This was checked through the respective root mean square errors (RMSE) and the determination coefficients for the plot of actual vs predicted pMAC (*q*^2^ and *r*^2^_test_ > 0.5 are considered acceptable). Also, since the MIA-QSAR model was obtained from PLS regression, a robustness test (*y*-randomization test) was performed to guarantee that calibration was not overfitted nor obtained by chance correlation; the *y* block was randomized and subsequently regressed against the intact matrix (ten times). Reliable models are achieved when *r*^2^_y-rand_ << *r*^2^, which is evaluated by ^c^*r*^2^_p_, defined as ^c^*r*^2^_p_ = *r* × (*r*^2^ − *r*^2^_y-rand_)^1/2^ [23]. Values above 0.5 for ^c^*r*^2^_p_ are considered acceptable. The statistical results for 9 latent variables (PLS components) illustrated in Figure 3 attest the predictability and reliability of the MIA-QSAR, thus suggesting that the 2D chemical structure indeed encodes biochemical properties and that MIA descriptors can be useful to anticipating pMAC of prospective congeneric drug-like candidates.

MIA-QSAR and 3D-QSAR have already been compared to each other in a variety of studies and the prediction results are similar [10,24–26], but the confidence in the interpretation provided by conformation-dependent methods is questionable as the minimum conformation may not necessarily represent the bioactive conformation. Both MIA-QSAR and 3D-QSAR methods are based on alignment rules and, therefore, they should be applied to congeneric series. Non-congeneric data sets are not within the applicability domain of MIA-QSAR and alignment-based methods and, since the alignment does not apply for these types of sets, the conformational landscape would affect only some specific descriptors used in classical QSAR, e.g., the 3D matrix-based descriptors available in the Dragon software.

**Figure 4:**
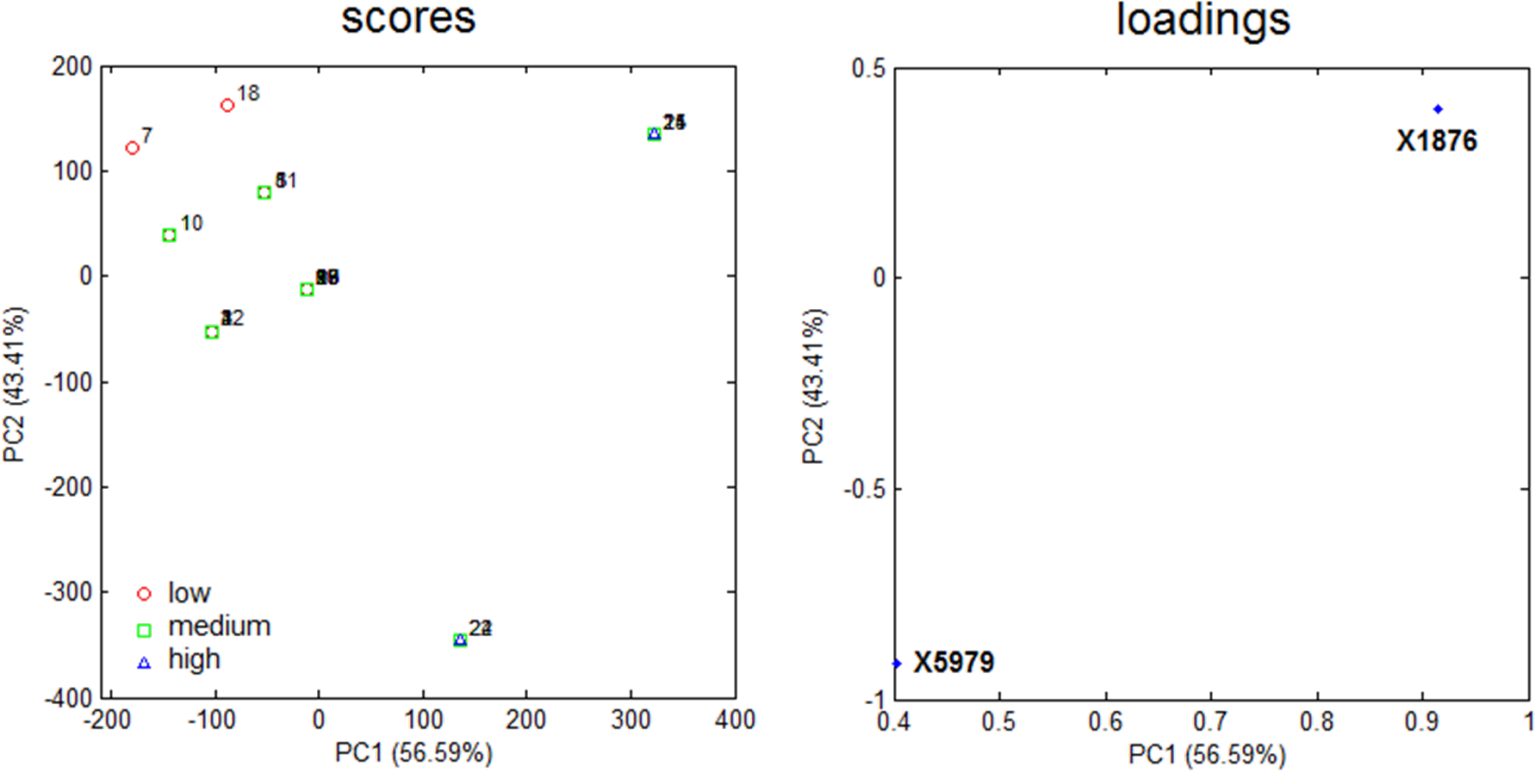
PCA plots for the mean-centered data of the 25 anesthetic haloethers.

Thus, a major goal in QSAR is to determine and interpret the chemical motifs/properties responsible for the observed biological effects. We have checked that even the simple log *P* model yields good prediction ability upon selection of test set compounds using the Kennard–Stone sampling (*r*^2^_test_ = 0.80); however, the main drawback of this analysis lies on the vague notion of the group types and/or molecular positions that most affect the pMAC values. Thus, the MIA descriptors were searched as source of chemical interpretation for the QSAR model. Because of the numerous MIA descriptors generated to build the model, a straightforward analysis would not be an easy task using the raw data. Thus, pre-filtration procedures were performed in order to reduce the number of variables. In this sense, the first approach was a measure of Shannon's entropy (SE), corresponding to an unsupervised classification variables filtering [27–28] applied to a 25 discrete intervals scheme. Variables with less than 10% of the maximum SE (SE_MAX_ = log 25) were discarded. The variables were filtered again through the correlation coefficient (x/x), with variables removed for each set of variables with a x/x = 0.98 [29]. As the data set under study is highly correlated, only two variables (X1876 and X5979) were identified containing further information regarding the anesthetic activity (pMAC). A careful analysis of the reduced matrix (Table 6) reveals the approximate positions of pixels X1876 and X5979 in the structural scaffold. Therefore, principal component analysis (PCA) was applied to obtain information on how these coordinates affect the bioactivity in terms of the two principal components PC1 (56.59%) and PC2 (43.41%) (Figure 4). Since PCA is a pattern recognition tool, the set of compounds were classified in three levels of anethestic intensity: low (pMAC ≤ 1.00 ), medium (1.00 < pMAC < 2.00), and high (pMAC ≥ 2.00) levels.

**Table 6:** Reduced matrix with the selected descriptors and their approximate coordinates in the images.

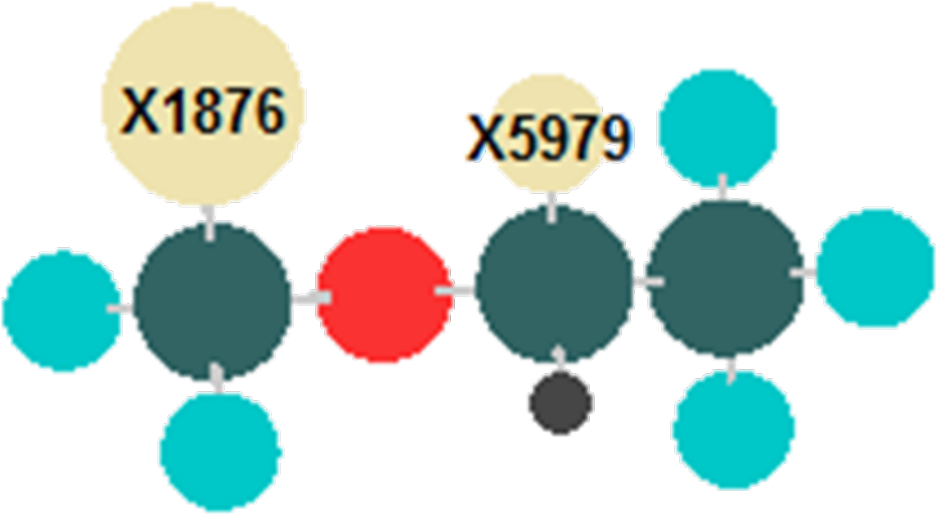		

Compound	X1876	X5979

**1**	300	300
**2**	300	400
**3**	300	400
**4**	300	400
**5**	400	300
**6**	400	400
**7**	300	210
**8**	400	300
**9**	400	400
**10**	300	300
**11**	400	300
**12**	300	400
**13**	400	400
**14**	765	400
**15**	400	400
**16**	400	400
**17**	400	400
**18**	400	210
**19**	400	400
**20**	400	400
**21**	765	400
**22**	400	765
**23**	400	400
**24**	400	765
**25**	765	400

From the PCA scores plot shown in Figure 4, three clusters are observed: one at positive scores in PC1 (compounds with moderate-to-high activities), another with negative scores in PC1 and around null scores in PC2 (compounds with moderate-to-low activities), and the third one at negative scores in PC1 and very positive scores in PC2 (compounds with low activities). Variables X1876 and X5979 in the loadings plot are responsible for clustering these compounds according to the scores plot. X5979 clearly separates compounds **22** (moderate activity) and **24** (high activity) from the remaining ones in PC2, due to their high pixel value at this position (765, white – blank space in the image). Bulk and hydrophobic substituents (R) at position R^4^ (surrounding X5979, see substituent numbering in Table 4) have long C–R bonds and, therefore, the pixel variable is located where small substituents (such as hydrogen, fluorine and even chlorine) appear, while the bromine atom does not occupy this coordinate because of its long bond distance with carbon. From this, since compounds **22** and **24** contain a bromine substituent at position R^4^ and pertain to the moderate-to-high activity class, such an halogen tends to favor an improved anesthetic activity of congeneric haloethers in this position.

PC1 explains most of the data variance and, therefore, variable X1876 should play a significant role for the bioactivity pattern of the data set compounds, because of its high loading in PC1. In addition to **22** and **24**, compounds **14**, **21** and **25** (also pertaining to the moderate-to-high activity class) lie in the region with positive scores in PC1, while, e.g., compounds **7** and **18** (low activity compounds) have very negative scores in PC1. In this case, the pixel value 765 at position X1876 indicates the absence of a substituent (hydrogen at R^1^) and, consequently, atoms encoded by high pixel values (e.g., 400 for the small and electronegative fluorine atom, but mainly for the small hydrogen atom) at R^1^ tend to strongly favor the increase in pMAC of anesthetic derivatives.

Because the absence of intermolecular hydrogen bonds between enflurane (a similar anesthetic haloether) and the integrin LFA1 enzyme [16], the structural requirements mentioned above should be dictated by hydrophobic interactions of R^4^ with amino acid residues and by low steric effects surrounding R^1^. Thus, 2D structural information provided by MIA descriptors (particularly related to the connectivity of different atoms in the present case) was capable of modeling and interpreting biochemical information of a series of anesthetic haloethers, without considering the conformation.

## Conclusion

It has been shown that a predictive and biochemically interpretable QSAR model can be obtained through bidimensional descriptors. Conformational insights could refine the analysis, but the risk of using wrong conformations could cause misinterpretation of the results. This eminent risk was assessed by investigating the conformational isomerism of isoflurane, whose bioconformation in at least one biological target is significantly different from the geometries optimized in a biological-free environment. A more secure option would be to obtain the ligand geometries inside an enzyme active site. Since conformational search inside a receptor normally gives the mode of interaction between substrate and enzyme, as well as the intermolecular interaction energy (related to the ligand–receptor affinity and, consequently, to the bioactivity), further QSAR analysis would be of limited utility.
